# Supplementary material for: Inflammation triggers immediate rather than progressive changes in monocyte differentiation in the small intestine
Source: Nat Commun. 2019 Jul 19;10:3229. doi: 10.1038/s41467-019-11148-2 (PMC6642215; doi:10.1038/s41467-019-11148-2)
Supplement: Supplementary file 1 — Supplementary Information [file 41467_2019_11148_MOESM1_ESM.pdf]

Supplementary Information

Inflammation triggers immediate rather than progressive changes in monocyte differentiation in the small intestine

Desalegn and Pabst

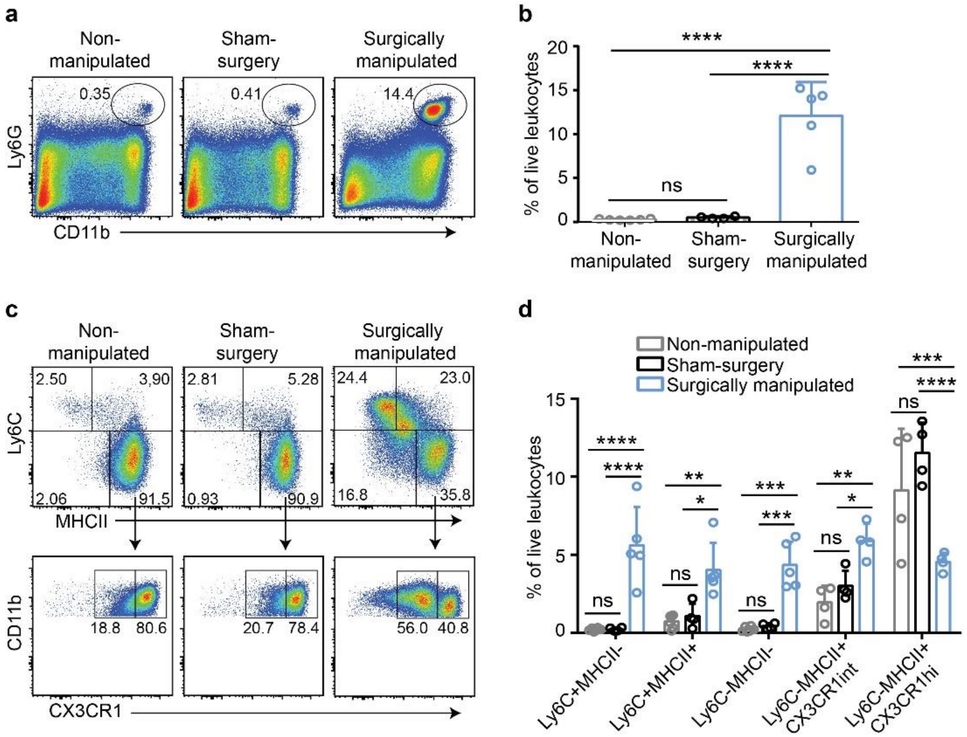

Supplementary Figure 1 | Intestinal manipulation, but not sham surgery, induces inflammation in the small intestine. CX3CR1$^{gfp/+}$ mice were left untouched (non-manipulated), underwent laparotomy (sham surgery) or underwent laparotomy followed by mechanical manipulation of the small intestine (surgically manipulated). Small intestinal myeloid cells were analyzed 2 days after the procedure (**a**) Representative FACS plots showing the frequency of CD11b$^+$Ly6G$^+$ neutrophils among live leukocytes in non-manipulated, sham surgery and surgically manipulated mice. (**b**) Frequency of CD11b$^+$Ly6G$^+$ neutrophils in small intestinal lamina propria. Data were pooled from at least 2 independent experiments with a total of at least 5 mice each. One-way ANOVA followed by Sidak's multiple comparison tests; mean±s.d.; ns, not significant; **** $p<0.0001$. (**c**) Representative FACS plots showing expression of Ly6C, MHCII and CX3CR1/GFP among endogenous CD64$^+$CD11b$^+$Ly6G$^-$ cells (MNP^{CD64+}). (**d**) Frequency of respective subsets of MNP^{CD64+} cells in small intestinal lamina propria of non-manipulated, sham surgery and surgically manipulated CX3CR1$^{gfp/+}$ mice. Data were pooled from at least 2 independent experiments with a total of at least 4 mice each. Two-way ANOVA followed by Sidak's multiple comparison tests; mean±s.d.; ns, not significant; * $p<0.05$, ** $p<0.01$, *** $p<0.001$, **** $p<0.0001$.

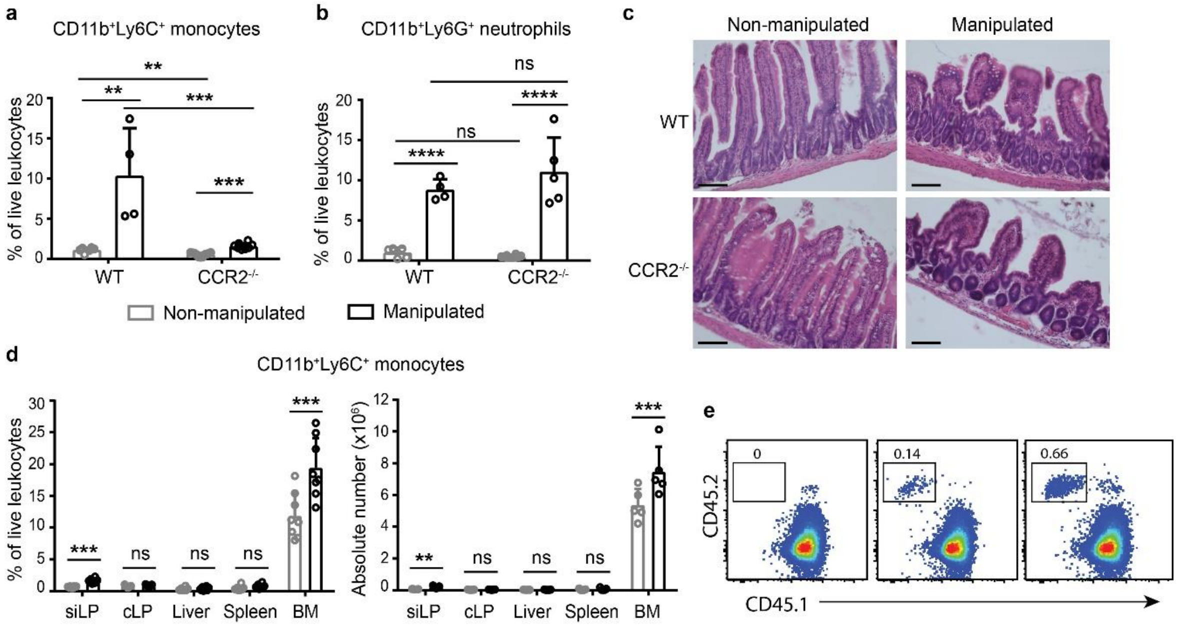

Supplementary figure 2│ Small intestinal manipulation induces inflammation in the small intestine of WT and CCR2$^{-/-}$ mice. (**a-d**) Small intestines of WT and CCR2$^{-/-}$ mice were surgically manipulated and 2 days later, mice were sacrificed for analysis. Frequency of CD11b^{+}Ly6C^{+} endogenous monocytes **(a)** and CD11b^{+}Ly6G^{+} neutrophils (**b**) among live leukocytes in small intestines of non-manipulated and manipulated WT and CCR2$^{-/-}$ mice. Data were pooled from at least 2 independent experiments with a total of at least 4 mice each. Two-way ANOVA followed by Sidak's multiple comparison tests; mean±s.d.; ns, not significant; * p<0.05, ** p<0.01, *** p<0.001, **** p<0.0001. (**c**) H&E staining of paraffin section taken from jejunum of non-manipulated and manipulated WT and CCR2$^{-/-}$ mice (Scale bar, 100µm). (**d**) Frequency among live leukocytes and absolute number of CD11b^{+}Ly6C^{+} endogenous monocytes in small intestine, liver, spleen and BM of non-manipulated and manipulated CCR2$^{-/-}$ mice. Data were pooled from at least 3 independent experiments with a total of at least 5 mice each. Unpaired t-test; mean±s.d.; ns, not significant; *** p<0.001. (**e**) Representative FACS plot of donor cells among live CD11b^{+} cells isolated from small intestine of non-manipulated WT (middle panel) and CCR2$^{-/-}$ (right panel) recipients one day after transfer and PBS injected control (left panel). Data are representative of at least 2 independent experiments.

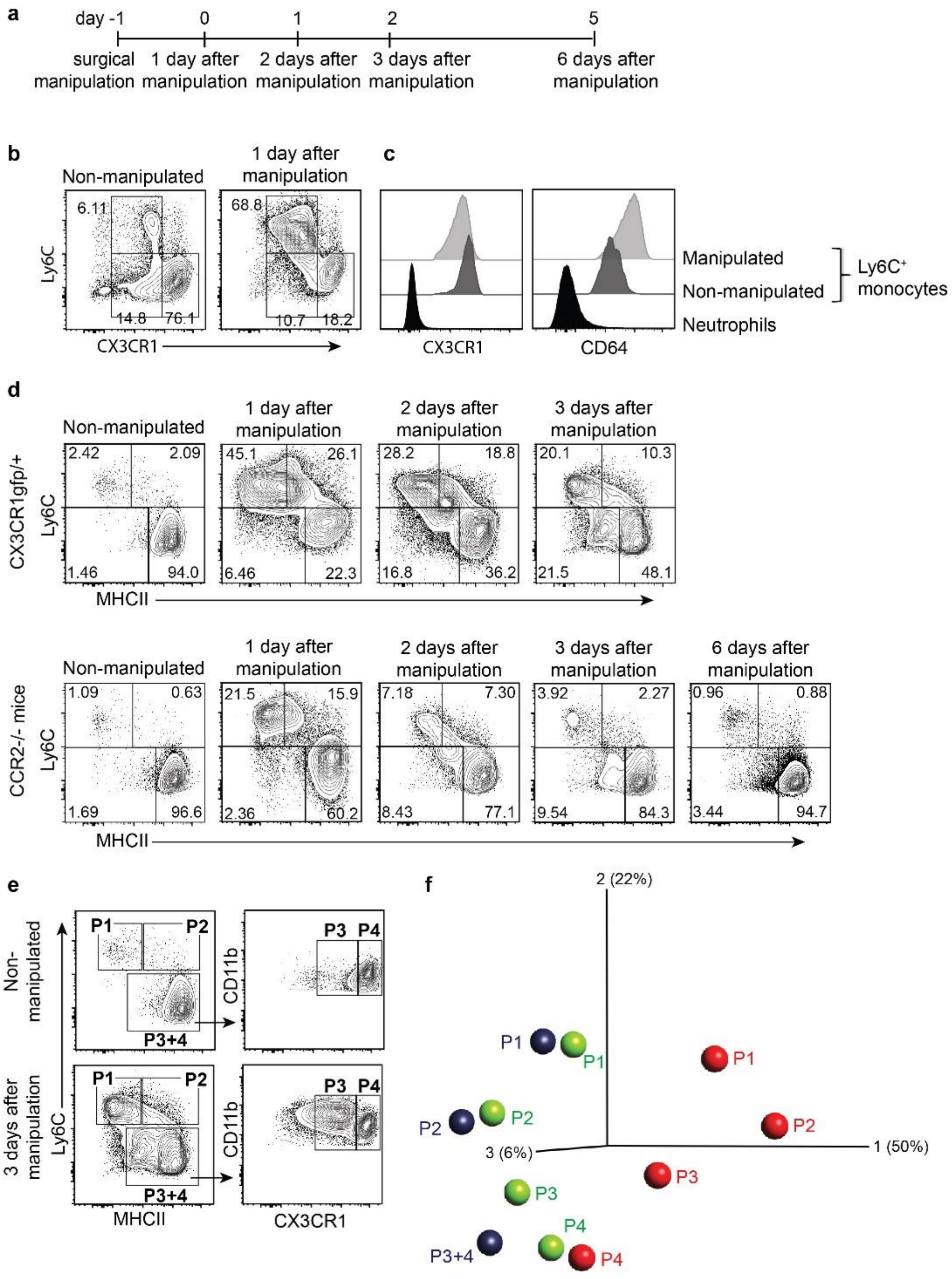

Supplementary figure 3 | Endogenous populations of MNPs in CCR2-deficient mice resemble the phenotypes observed in WT mice. (**a**) Schematic illustration of the experimental set-up. Please note that we label the day of manipulation as '-1' to align panels displayed in this figure with figures 3 and 4. (**b**) Phenotypic comparison of intestinal MNP^{CD64+} cells from non-manipulated and manipulated mice. One day after surgical manipulation of the small intestine in CX3CR1$^{gfp/+}$ mice, MNP^{CD64+} cells were characterized based on expression of Ly6C and CX3CR1/GFP and compared to cells isolated from non-manipulated mice. (**c**) Representative histograms demonstrating expression of CX3CR1 and CD64 on Ly6C^{+}CX3CR1^{int}

monocytes (as gated in **b**) and neutrophils (CD11b$^+$Ly6G$^+$) for comparison. (**d**) Representative FACS plots comparing MNP^{CD64+} populations between CX3CR1$^{gfp/+}$ mice and CCR2-deficient mice. Population frequencies differ between both mouse strains. However, qualitatively the changes in the populations from CCR2-deficient mice resemble the kinetics observed in CX3CR1$^{gfp/+}$ mice. (**e**) Sorting strategies for MNP^{CD64+} cells from CCR2-deficient and CX3CR1$^{gfp/+}$ mice. Endogenous MNP^{CD64+} cells were sorted to obtain populations P1, P2, P3 and P4 as indicated from non-manipulated CX3CR1$^{gfp/+}$ mice and 3 days after manipulation. For comparison populations P1, P3 and P3+P4 were obtained from non-manipulated CCR2-deficient mice. **(f**) The populations sorted in **e** were analyzed by nanostring analysis. PCA analysis reveals a higher similarity between P1, P2, and P3+P4 comparing non-manipulated CX3CR1$^{gfp/+}$ (green symbols) and CCR2-deficient mice (blue symbols) as compared to manipulated CX3CR1$^{gfp/+}$ mice (red symbols). Data in **b-e** are representative of at least 2 independent experiments. Data in **f** depict a single experiment.

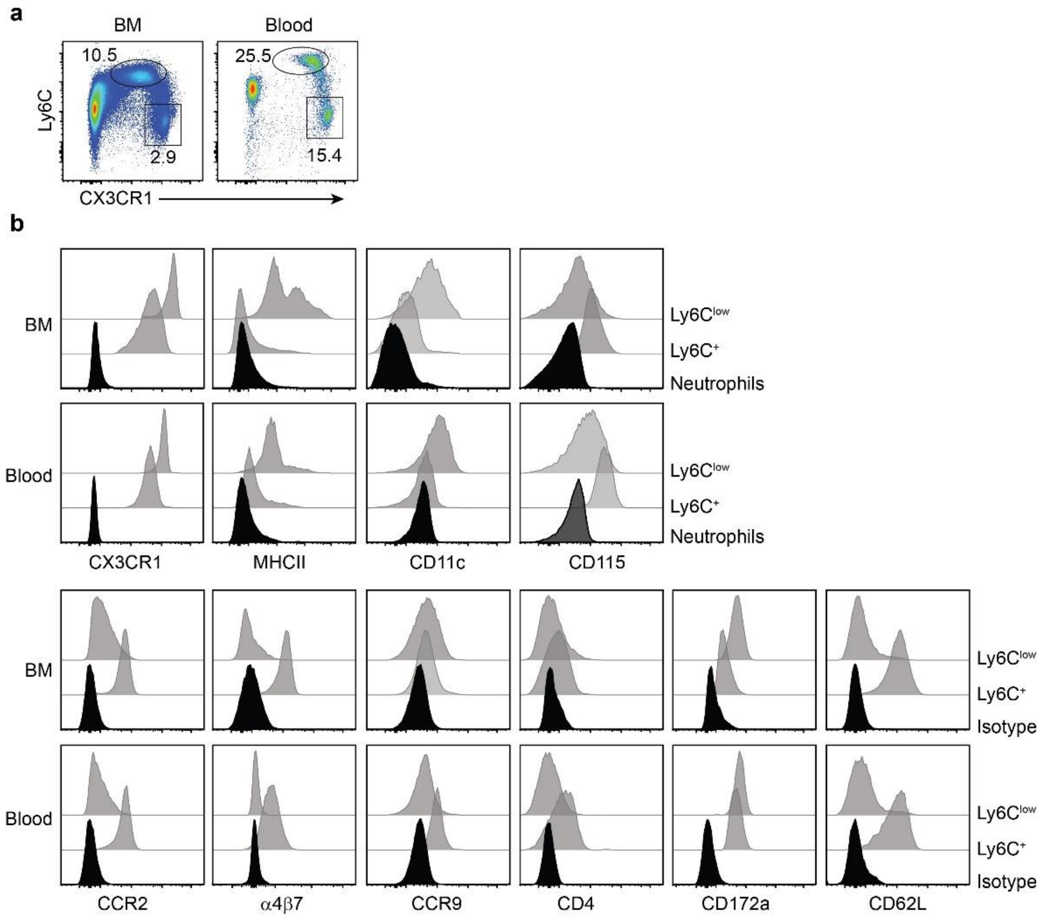

Supplementary Figure 4 | Ly6C^{+} monocytes appear as a homogeneous population in BM and blood. (**a**) Representative FACS plots demonstrating Ly6C^{+} and Ly6C^{-} monocytes after gating on live CD11b^{+} leukocytes in the BM and blood of CX3CR1$^{gfp/+}$ mice. (**b**) Phenotyping of Ly6C^{+} cells. Top, expression of CX3CR1/GFP, MHCII, CD11c and CD115 on Ly6C^{+} and Ly6C^{low} monocytes compared to endogenous neutrophils. Bottom, expression of CCR2, $\alpha 4\beta 7$, CCR9, CD4, CD172a and CD62L on Ly6C^{+} and Ly6C^{low} monocytes and isotype controls. Data are representative of at least 2 independent experiments with at least 4 mice each.

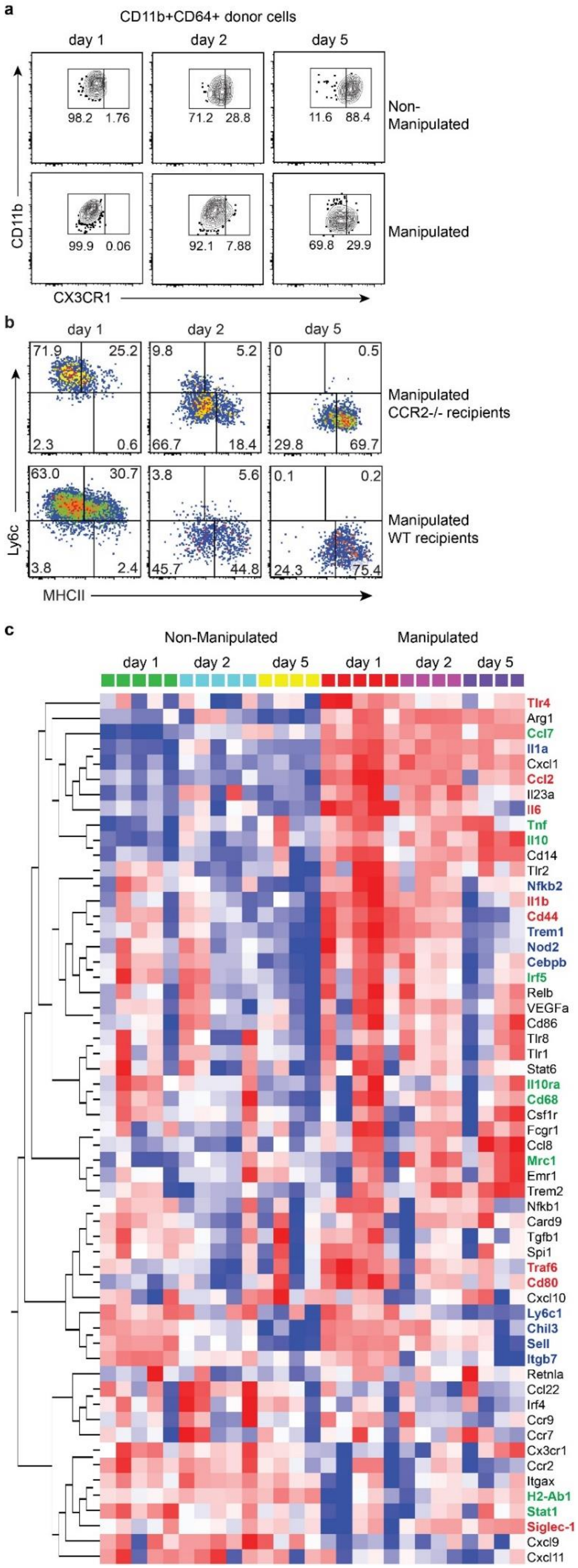
a
CD11b+CD64+ donor cells
day 1
day 2
day 5
98.2 1.76
71.2 28.8
11.6 88.4
Non-Manipulated
99.9 0.06
92.1 7.88
69.8 29.9
Manipulated
CD11b
CX3CR1
b
day 1
day 2
day 5
71.9 25.2
2.3 0.6
9.8 5.2
66.7 18.4
0 0.5
29.8 69.7
Manipulated CCR2-/- recipients
63.0 30.7
3.8 2.4
3.8 5.6
45.7 44.8
0.1 0.2
24.3 75.4
Manipulated WT recipients
Ly6c
MHCII
c
Non-Manipulated
Manipulated
day 1
day 2
day 5
day 1
day 2
day 5
Tlr4
Arg1
Ccl7
Il1a
Cxcl1
Ccl2
Il23a
Il6
Tnf
Il10
Cd14
Tlr2
Nfkb2
Il1b
Cd44
Trem1
Nod2
Cebpb
Irf5
Relb
VEGFa
Cd86
Tlr8
Tlr1
Stat6
Il10ra
Cd68
Csf1r
Fcgr1
Ccl8
Mrc1
Emr1
Trem2
Nfkb1
Card9
Tgfb1
Spi1
Traf6
Cd80
Cxcl10
Ly6c1
Chil3
Sell
Itgb7
Retnla
Ccl22
Irf4
Ccr9
Ccr7
Cx3cr1
Ccr2
Itgax
H2-Ab1
Stat1
Siglec-1
Cxcl9
Cxcl11

Supplementary Figure 5 | Inflammation alters monocyte differentiation program in the small intestine. (**a**) CX3CR1 expression of donor MNP^{CD64+} cells isolated from the small intestine of non-manipulated and manipulated CCR2-dificient recipients at day 1, 2 and 5 post-transfer. (**b**) Representative FACS plots comparing phenotypic changes of donor MNP^{CD64+} cells over time, with respect to their expression of Ly6C and MHCII. The upper panel demonstrates the phenotype of MNP^{CD64+} cells in CCR2-deficient recipients (data depicted are identical to Fig. 4e and are shown here for direct comparison), the lower panel shows the phenotype in WT recipients. (**c**) Heat map demonstrating the expression profile of all detectable genes that were included in the nanostring analysis in adoptively transferred MNP^{CD64+} cells that were recovered small intestinal lamina propria (siLP) of non-manipulated and manipulated recipients at day 1, 2 and 5 after transfer. Genes that were differentially expressed at different time points in non-manipulated recipients are labeled green, in manipulated recipients are labeled red, and in both manipulated and non-manipulated recipients are labeled blue (cut-off *p* value is 0.05). Genes typed in black font did not differ significantly in their expression.

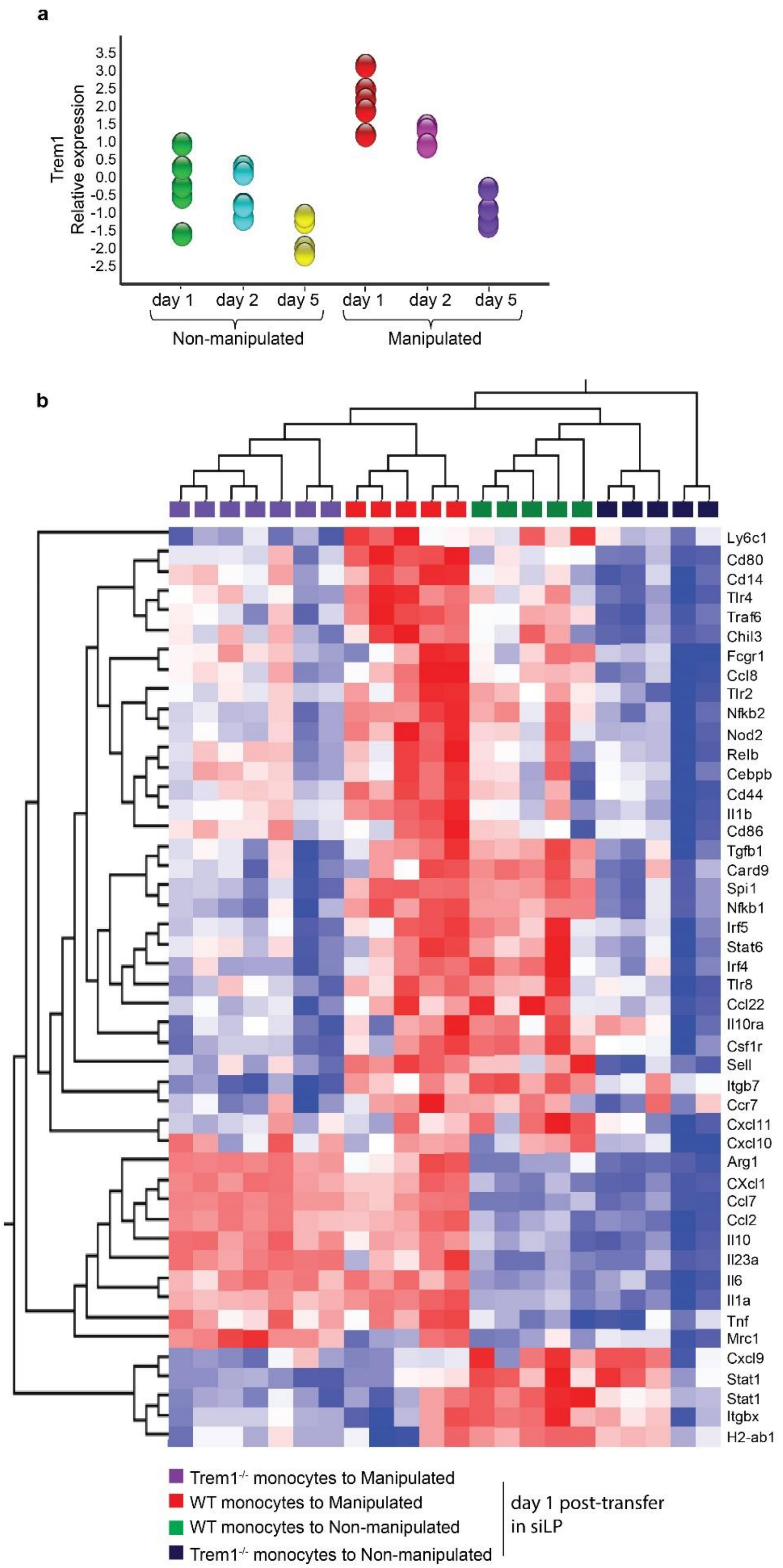
a
Trem1
Relative expression
3.5
3.0
2.5
2.0
1.5
1.0
0.5
0.0
-0.5
-1.0
-1.5
-2.0
-2.5
day 1
day 2
day 5
day 1
day 2
day 5
Non-manipulated
Manipulated
b
Ly6c1
Cd80
Cd14
Tlr4
Traf6
Chil3
Fcgr1
Ccl8
Tlr2
Nfkb2
Nod2
Relb
Cebpb
Cd44
Il1b
Cd86
Tgfb1
Card9
Spi1
Nfkb1
Irf5
Stat6
Irf4
Tlr8
Ccl22
Il10ra
Csf1r
Sell
Itgb7
Ccr7
Cxcl11
Cxcl10
Arg1
CXcl1
Ccl7
Ccl2
Il10
Il23a
Il6
Il1a
Tnf
Mrc1
Cxcl9
Stat1
Stat1
Itgbx
H2-ab1
Trem1-/- monocytes to Manipulated
WT monocytes to Manipulated
WT monocytes to Non-manipulated
Trem1-/- monocytes to Non-manipulated
day 1 post-transfer
in siLP

Supplementary Figure 6 | Small intestinal inflammation shapes the transcriptomic signature of incoming monocytes in a partially Trem1-dependent mechanism (**a**) Relative expression of Trem-1 determined by the nanostring analysis depicted in Supplementary Figure 5b. Expression was normalized according to the mean expression observed in non-manipulated recipients one day after transfer. (**b**) Heat map showing hierarchal clustering of gene expression profiles of adoptively transferred MNP^{CD64+} WT or Trem-$1^{-/-}$ cells. Donor monocyte-derived cells were recovered from from small intestinal lamina propria (siLP) of recipient mice one day after adoptive transfer. Recipients were surgically manipulated one day before adoptive transfer (manipulated recipients) or non-manipulated. Gene expression were determined by nanostring nCounter analysis and differentially expressed genes were determined by Qlucore Omics explorer with a statistical significance *p* value of 0.05. Data for WT cells are identical to data depicted in for day 1 in Supplementary Figure 5b. Additionally, Trem1-deficient monocytes were analyzed one day after transfer into manipulated recipients or non-manipulated recipients and compared to WT monocytes. Findings in Trem1 deficient cells were pooled from 2 independent experiments with a total of 5-7 recipients analyzed.

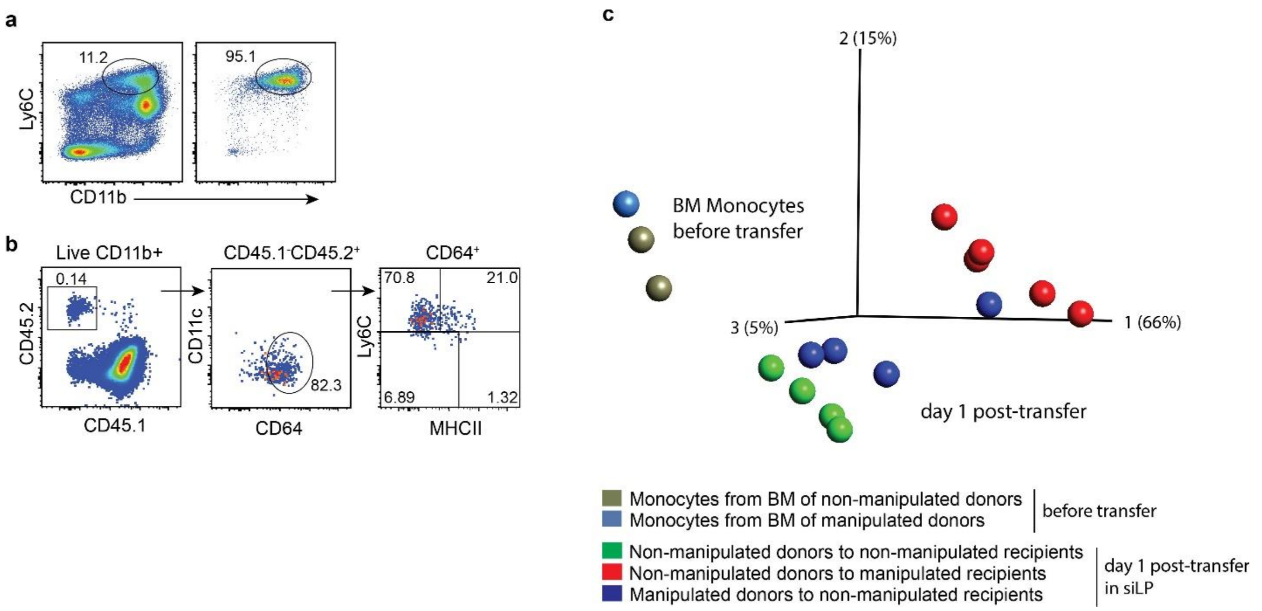

Supplementary Figure 7 | Small intestinal inflammation of recipient but not of monocyte donor mice profoundly affects the phenotype of monocytes entering the intestine. (**a**) Representative FACS plots demonstrating frequencies of $CD11b^+Ly6C^+$ monocytes among live leukocytes in the BM of manipulated donor WT mice before (left panel) and after enrichment (right panel). BM cells were isolated and $CD11b^+Ly6C^+$ monocytes were purified one day after manipulation of the small intestine of donor mice. Compare with Fig. 1a. (**b**) Transferred cells were identified by congenic markers (left panel) and the donor monocytes identified as $CD11b^+CD11c^{low/int}CD64^+$ cells (middle panel) from small intestinal lamina propria (siLP) of recipient mice. Donor MNP^{CD64+} cells were further characterized based on Ly6C and MHCII expression (right panel). **(c)** PCA representing transcriptomic profiles of purified BM monocytes from non-manipulated (light grey) and manipulated (light blue) donors, and adoptively transferred MNP^{CD64+} cells recovered from siLP of non-manipulated (green) and manipulated (dark blue) recipients (red) at day 1 post-transfer. Donor cells derived from manipulated and non-manipulated donors were more similar to each other as compared to monocytes recovered from manipulated recipients. Symbols represent individual mice analyzed. Qlucore Omics explorer was used for gene expression analysis and a statistical significance *p* value of 0.05 was used to determine differentially expressed genes.

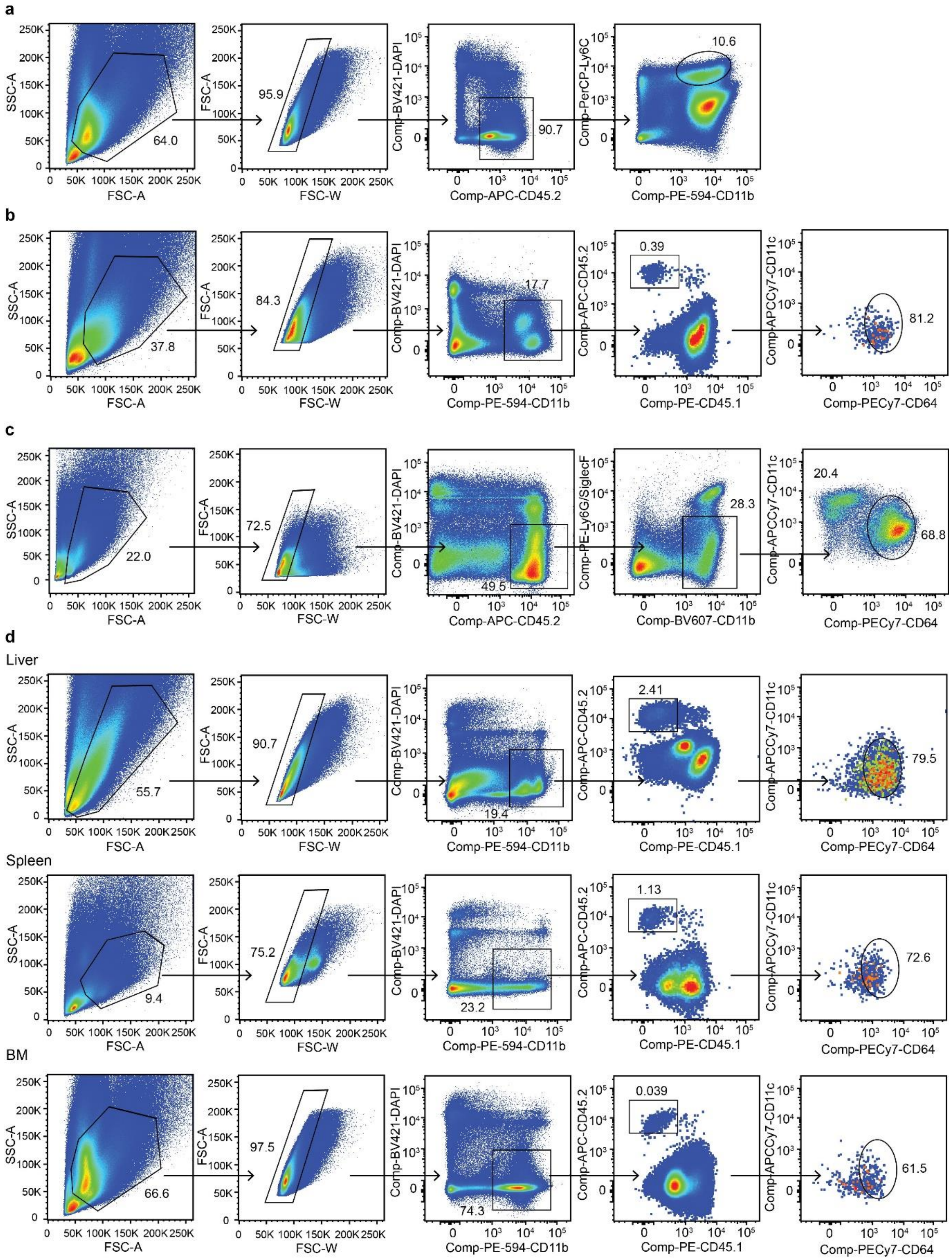

Supplementary Figure 8 | FACS gating strategies. (**a**) Representative plots showing the gating strategy of BM cells to identify CD11b^{+}Ly6C^{+} monocytes (used in Figure 1a). (**b**) Representative plots showing the gating strategy used to recover and sort donor monocyte-derived MNP^{CD64+} cells from the small intestine

of recipients (used in Figure 1d, 2c, 3d and 4d). (**c**) Representative plots showing the gating strategy used to define endogenous MNP^{CD64+} cells from small intestine of $CX3CR1^{gfp/+}$ mice (used in Figure 3a and 4a). (**d**) Representative plots showing the gating strategy used to recover and sort donor monocyte-derived MNP^{CD64+} cells from the liver, spleen and BM of recipients (used to generate data displayed in Figure 6).

Supplementary Tables

Supplementary Table 1: List of genes and sequences of respective MTE primers

Genes	NSID (Accession #)	Primers	
		**Forward Sequence**	**Reverse Sequence**
Actb*	NM_007393.1:815	TATGAGCTGCCTGACGGC	CAGCAATGCCTGGGTACA
Arg1	NM_007482.3:626	CTGGGTGACTCCCTGCAT	AGCCCGTCGACATCAAAG
Card9	NM_001037747.1:1227	GCTGGGTGAGAAGGCAGA	TCTGAGAGCTGGGCATCC
Ccl2	NM_011333.3:415	ACCTGGATCGGAACCAAA	GGCATCACAGTCCGAGTCA
Ccl22	NM_009137.2:1096	GGAATCTTTCTCCCATCCCT	GCAGTCACTGACACCATAGCA
Ccl7	NM_013654.2:215	AATGCATCCACATGCTGC	AAGGCTTTGGAGTTGGGG
Ccl8	NM_021443.2:150	CCCTGTCAGCCCAGAGAA	CCCACTTCTGTGTGGGGT
Ccr2	NM_009915.2:2965	TGCCAAGGTTGAAAATAGTTCAT	TTGCATGCACACATGACATC
Ccr7	NM_007719.2:755	TACAGCGGCCTCCAGAAG	ACCACGGCAATGATCACC
Ccr8[#]	NM_007720.2:426	GTGGGTGTTTGGGACTGC	ATCAAGGGGATGGTGGCT
Ccr9	NM_009913.6:820	GGGGTTTTTCCTCCCCTT	GCGTCAACAGCCTGCAC
Cd14	NM_009841.3:235	CGTGTGCTTGGCTTGTTG	CCCAGATCTGCTTCCGTG
Cd163[#]	NM_053094.2:3225	TCCAGTGCCTCCCAAAAA	CCAGATCATCCGCCTTTG
Cd44	NM_001039150.1:2475	CAGGGAACATCCACCAGC	TAGCATCACCCTTTGGGG
Cd68	NM_009853.1:636	ACCCATCCCCACCTGTCT	GCTTGCATTTCCACAGCA
Cd80	NM_009855.2:210	GCCTAAGCTCCATTGGCTC	ACGGCAAGGCAGCAATAC
Cd86	NM_019388.3:251	ATCTGCCGTGCCCATTTA	ACGAGCCCATGTCCTTGA
Cebpb	NM_009883.3:1147	TGCACCGAGGGGACAC	AACCCCGCAGGAACATCT
Chil3	NM_009892.1:1196	GGGAGATCTCAATATACACAGTGC	CCCAGCTGGTACAGCAGAC
Csf1r	NM_001037859.2:1892	CCCCGATGAGTCCCTCTT	CCGAGGGAACTCCCACTT
Csf2[#]	NM_009969.4:452	GCCCTGAACCTCCTGGAT	GCTGTCATGTTCAAGGCG
Cx3cl1[#]	NM_009142.3:125	TTCCTGCACTCCAGCCAT	TGCTGTGTCGTCTCCAGG
Cx3cr1	NM_009987.3:2696	GGTCCCAAGTTCAATTCCC	TTGGATATGCCTGTGGGG
Cxcl1	NM_008176.1:560	TTGGGAGACCACTAAGTGTCAA	AAGGGAAGCGTCAACACG
Cxcl10	NM_021274.1:115	TGCCGTCATTTTCTGCCT	CGTGGCAATGATCTCAACA
Cxcl11	NM_019494.1:345	CGACAAAGGTGCCTGGAC	GCCTGGTAATACGTGGCTG
Cxcl9	NM_008599.2:40	TCGGACTTCACTCCAACACA	TTGTTGCAATTGGGGCTT
Emr1	NM_010130.1:995	CACCCTGGCTTTGCATCT	TTCCATATCCTTGGGAGCC
Fcgr1	NM_010186.5:185	TCACCTTGCAGCCTCCAT	TTGCAACTGCACAGGGTC
Flt3[#]	NM_010229.2:3302	GCCAAGATTAGCCTCGCC	ATGGCTTTCCCCTCAAGC
Gapdh*	NM_001001303.1:890	TCTGAGGGCCCACTGAAG	GCCATGTAGGCCATGAGG

Gusb*	NM_010368.1:1735	CGCCTCGCATGTTCAGT	AAAGGCCGAAGTTTTGGG
H2-Ab1	NM_207105.2:164	GTGGTGCTGATGGTGCTG	CAGTACTCGGCGTCTGGC
Hprt*	NM_013556.2:30	GCAGCGTTTCTGAGCCAT	CATCGCTAATCACGACGC
Il10	NM_010548.1:985	AGGCTGGCCACACTTGAG	GGGGGATGACAGTAGGGG
Il10ra	NM_008348.2:75	GTAAAGGCCGGCTCCAGT	TGGAGGATGTGCTGGAAAA
Il1a	NM_010554.4:225	GGCTCACTTCATGAGACTTGC	GGTGCTGATCTGGGTTGG
Il1b	NM_008361.3:1120	CCTCTGATGGGCAACCAC	TGTGCTGGTGCTTCATTCA
Il23a	NM_031252.1:360	GCCCCGTATCCAGTGTGA	GCTGGAGGAGTTGGCTGA
Il4#	NM_021283.1:345	AGGGCTTCCAAGGTGCTT	TGCTCTTTAGGCTTTCCAGG
Il6	NM_031168.1:40	CAATTCCAGAAACCGCTATGA	TCATTTCCACGATTTCCCA
Irf4	NM_013674.1:1878	CAGAGAAACGCATTCCTGG	AGTCCACCAGCTGGCTTTT
Irf5	NM_012057.3:1826	GTGGGAGGGAGGGAACAT	TCTGAAGGAGGGTGCCTG
Itgax	NM_021334.2:327	ACAGGCAACTGTGAGCCC	TGCTGTGCAGTTGGGAAG
Itgb7	NM_013566.2:1221	TGTCACCGGTGCAACACT	AGCCTCACCCTCCGTCTT
Ly6c1	NM_010741.2:236	TGAGACTTCCTGCCCAGC	GGTGCTACCTGCAGTGGG
Mrc1	NM_008625.1:3992	TCCCTGGTTTCCATCGAG	CAAAGGCCCGAAGATGAA
Nfkb1	NM_008689.2:2125	GCCCTTTTCGACTACGCA	GGCCAAGTGCAGAGGTGT
Nfkb2	NM_019408.2:1150	GCAAGCCTTTGGGGACTT	GCCTTTCTCCGCTTCCTC
Nod2	NM_145857.2:1086	TTTGAGCACTGCTGCTGG	GCACCTTGCAGGCATTCT
Nos2#	NM_010927.3:3715	CTTGGGGAGACAGCGAAA	GAGAAGGAGGGAGCTGGG
Pparg#	NM_011146.1:1060	TTCGATCCGTAGAAGCCG	GCTTCCGCAGGTTTTTGA
Relb	NM_009046.2:2013	TTCCGAAGCCAACCTTGA	TTAGGGGAGAAGGCTGGG
Retnla	NM_020509.3:164	TCTGCATCTCCCTGCTCC	AGGCAAAGCCACAAGCAC
Rpl19*	NM_009078.2:92	GGCCCACAAGCTCTTTCC	AGTCACAGGCTTGCGGAT
Sell	NM_001164059.1:664	GACTGCATCCACCCCTTG	GGTACCCAACTCAGGGGC
Siglec1	NM_011426.3:4550	GCCTGGTTTCACAATGGC	CCATGAGCCTGGTCCTTG
Spi1	NM_011355.1:171	CGAAGCAGGGGATCTGAC	GTGTGCGGAGAAATCCCA
Stat1	NM_009283.3:1590	TCCGACACCTGCAACTGA	ATAGACGCCCAGCCACTG
Stat3#	NM_213659.2:2130	TTCAGCGAGAGCAGCAAA	AAATGCCTCCTCCTTGGG
Stat6	NM_009284.2:3465	TGCTTTTGCCAGTGTGACC	ACGCCCAGGGAGTTTACA
Tgfb1	NM_011577.1:1470	CGCCTGAGTGGCTGTCTT	ATGTCATGGATGGTGCCC
Tlr1	NM_030682.1:805	TCGGCACGTTAGCACTGA	TCCACGTTGTTTCCACATTG
Tlr2#	NM_011905.2:255	TCCCCTTCCCTCACTTCC	GAAACAGTCCGCACCTCC
Tlr4	NM_021297.2:2510	GCCTCGAATCCTGAGCAA	CCTTCTGCCCGGTAAGGT
Tlr8	NM_133212.2:110	TCTGGAACCAGTGCCATCT	TGAGGTTTTGCAGCTTTTGA

Tnf	NM_013693.1:1135	GTGACCTGGACTGTGGGC	TGGCTCTGTGAGGAAGGC
Traf6	NM_009424.2:980	GTCCCAGGAGGCAGGTTT	AGCTGTTGGGCAGTCCAG
Trem1	NM_021406.5:1704	TGCCCCCTGTGTGAGTCT	TTGTGGTGATGCGGTTTG
Trem2	NM_031254.2:646	TGGAACACAGCACCTCCA	TACCTCCGGGTCCAGTGA
Vegfa	NM_009505.4:1830	AAGCACGGTCCCTCGTG	TCCAGTGAAGACACCAATAACA
Zbtb46	NM_028125.1:830	GGGAAGGAGGACCAGGAA	ATTCTTCCTCCGGCCTGT

*Housekeeping genes

#genes excluded from the analysis because a general low detection level

Supplementary Table 2: List of genes and sequences of respective probes

Genes	Probe A	Probe B
Actb*	AAAAGAGCCTCAGGGCATCGGAACCGCTCGTTGCCAATAGTGATGACCTGCAAAGACGCCTATCTTCCAGTTTGATCGGGAAACT	CGAAAGCCATGACCTCCGATCACTCATGTAGTTTCATGGATGCCACAGGATTCCATACCCAAGAAGGAAGGCTGG
Arg1	TTTTTATTATATAGTGTTCCCCAGGGTCTACGTCTCGCAAGCCAATGCGTGAACCAGATTATGTATGGACGCGCAATAGATA	CGAAAGCCATGACCTCCGATCACTCAATCCCCAGCTTGTCTACTTCAGTCATGGAGAAATACTTAATTCCCAGAG
Card9	TCAGAGCTCAGGATGAGCATGTCCAATTGCTGCTGCTTGAGCATTCGCAACCATGTGAAGTAATGTGAGCGTACTT	CGAAAGCCATGACCTCCGATCACTCAGAGCTCCTGGGAGTTCCTGGGTGAACTGTCTTCCAAG
Ccl2	GCATTAGCTTCAGATTTACGGGTCAACTTCACATTCAAAGGTGCTGAAGACACAAGAATCCCTGCTAGCTGAAGGAGGGTCAAAC	CGAAAGCCATGACCTCCGATCACTCTCACTCCTACAGAAGTGCTTGAGGTGGTTGTGGAAAAGGTAGTGGAT
Ccl22	AGTAAAAGACCCTAGCATGTGGTTGAAGAGGGGTGGAAGTTGATTCTTGGCCTATTGAAGCAATCCTCTCCCCAATACTTAAAAA	CGAAAGCCATGACCTCCGATCACTCCTTCGACTATCAGCTACACAGGCAAGGAGTCAAAGGTGTGGGGCAGAGAA
Ccl7	CTTGAAGATAACAGCTTCCCAGGGACACCGACTACTGGTGATCCTTCTGTCCACGCGATGACGTTCGTCAAGAGTCGCATAATCT	CGAAAGCCATGACCTCCGATCACTCCCACTTCTGATGGGCTTCAGCACAGACTTCCATGCCCTTCTTTGT
Ccl8	TTCAGCACCCGAAGGGGGATCTTCAGCTTTAGTACATGAAAGCAGCAGGTCATTTGGAATGATGTGTACTGGGAATAAGACGACG	CGAAAGCCATGACCTCCGATCACTCAAACCACAGCTTCCATGGGGCACTGGATATTGTTGATTCTCTCGTAGCTT
Ccr2	ACCACAAACCACAAGTCCCTGTTAATCCTGAGCTGTCTATGTTAGTTCATCTGTTGAGATTATTGAGCTTCATCATGACCAGAAG	CGAAAGCCATGACCTCCGATCACTCATCCAGACGGACCTTAGAGTCTAAATCACCATGCTTGGATAAGCCCACAG
Ccr7	GCAGAAACTCATAGCCAGCATAGGCACTAGGAACCCAAAAACCATCTGGGCTAGGACGCAAATCACTTGAAGAAGTGAAAGCGAG	CGAAAGCCATGACCTCCGATCACTCTTCCGCTCAAAGTTGCGTGCCTGGAGCAAGGTACGGATGATAATGAGGTA
Ccr8#	CGTGGACAATAGCCAGATACCTGTCCACACTCATTAGGGTGATGAAGAACCGCTTTATTATGTGTTCGTCTAACTCTGTTTCTGT	CGAAAGCCATGACCTCCGATCACTCCACGCTGGCCGTCCTCACCTTGATGGCATAGACAG
Ccr9	TGCTTGGATGACTTCTTGGCCTGTACCAAGGTATGAATGATGATGGTATACTGACACATTAGTAACGTCGGCAAGCACTTAGTCG	CGAAAGCCATGACCTCCGATCACTCGAGACATAATGAAGACAGTGAGGACAGTGATGGTCACCTTGAGGGCCTTG
Cd14	CAATCTGGCTTCGGATCTGAGAAGTTGCAGGAACAACTTTCCTCGTCTAGCTATTTCTGTTCACGGATGAAGGCCTATATCAATG	CGAAAGCCATGACCTCCGATCACTCCGCCGTACAATTCCACATCTGCCGCCCCAAACAATTGAAAGCGCTGGAC
Cd163#	GACAATGAGGAGGACCAATAGAATGGCTCCACAAACCAAGAGTGCCGTGACCTGGAGTTTATGTATTGCCAACGAGTTTGTCTTT	CGAAAGCCATGACCTCCGATCACTCGAAACTGTAAGTCGCTGAATCTGTCGTCGCTTCAGAGTCCACAGGAGGAA
Cd44	CCCCGCTGGAAAGCAATGCCTAAGGATTGGACTGCTGGAGAGGCAATATGCCATCCACTTTCATGGAAACAATAAGAGCAGGGAA	CGAAAGCCATGACCTCCGATCACTCCAGAAGGGAGTCTATAAAGAGAGTATGTAACCAGTACACCTGATCTCCCA

Cd68	GACACATTGTATTCCACCGCCATGTAGTCCAGGTAGACTGTACTCGCTTGAGCTCTAGGCCCAAAACGACCTTAATGGTCA	CGAAAGCCATGACCTCCGATCACTCGAAGAGATGAATTCTGCGCCATGAATGTCCACTGTGCTGCCTGTGGGAAG
Cd80	ATTGCAAGCCATAGCTTCAGATGCTTTGGAGAACATGATGGGGAAAGCCACTTCAGTTAAAGGCTATCTTGCTCCGCTCGTTCTC	CGAAAGCCATGACCTCCGATCACTCAGCCTTGGACATGGAAACTTGAGGAGTGGTGTATCCTGCATCAACTGACA
Cd86	TTTTGCTGGTCCTGCCAAAATACTACCAGCTCACTCAGGCTTATGCTTAAAGCTATCCACGAATGTCAAAAATGTGGTTT	CGAAAGCCATGACCTCCGATCACTCTCACACTATCAAGTTTCTCTGTGCCCAAATAGTGCTCGTACAGAACCAAC
Cebpb	TCAAGTCCCGAAACCCGGTGCGCTGTGCAGGTGCGTGCGCGCAGCAAGAAGGAGTATGGAACTTATAGCAAGAGAG	CGAAAGCCATGACCTCCGATCACTCCCGTGTCCACACGCGCTCAGCCACGTTTGATCCGGATTGCA
Chil3	TGATCTTATTCTGAGAGTTTCAAGGACAAATCATTGTGTAAAGCTCCTCTCATACGAAATTTGAGCAAGCAATTGAAGGCTTAGA	CGAAAGCCATGACCTCCGATCACTCTTCTCCATGGAAGCATGATGCAGAATGCGCTGTGGAAAAACCGTTGAACT
Csf1r	ATCTCTCGATGATCTTCCAGCGCACCTGGTACTTCCTAGCCCAGATCCTACGAGATGAGCTACGTAACTA	CGAAAGCCATGACCTCCGATCACTCGCAACTGAGTAGGGTCAATGAAGGTGTAGCTATTGCCTTCGT
Csf2#	GGTCTGCACACATGTTAGCTTCTTGAAGGAGAACTCGTTAGAGACGACTTCCCGAATGTATAATGCTGACGTTCTTGCTTTTGGC	CGAAAGCCATGACCTCCGATCACTCTTTGGTGAAATTGCCCCGTAGACCCTGCTCGAATATCTTCAGGCG
Cx3cl1#	CGTCATGCCGAGGTGCTGACCCGGCAGCAGAGTACACAAATGGAAGAACGCCGAGTGCATGAGCTGTCTTTCACATGATACATCG	CGAAAGCCATGACCTCCGATCACTCAAAGCCACTGGGATTCGTGAGGTCATCTTGTCGCACATGATTTCGCATTT
Cx3cr1	GCAACAGGCTGGGAGAGACTTCTGAAATGGTTATCACAACCAACACAGGACACAATTCTGCGGGTTAGCAGGAAGGTTAGGGAAC	CGAAAGCCATGACCTCCGATCACTCTCGACACAAACATACAGACTGCCAAGGAGATATCAGACATGCCGCCGTGA
Cxcl1	TAAACTCTATCTCTCTGCACTTCTTTTCGCACAACACCCTTCTACTAGCACTTGACGTAGATTGCTATCAGGTTACGATGACTGC	CGAAAGCCATGACCTCCGATCACTCACACAGCCTCCCACACATGTCCTCACCCTAATACATACAAAACATAATAC
Cxcl10	ATTCTCACTGGCCCGTCATCGATATGGATGCAGTTGCAGCGCTCTGTGAACTGTCATCGGTCCGATCAATTAGTCT	CGAAAGCCATGACCTCCGATCACTCCTCGCAGGGATGATTTCAAGCTTCCCTATGGCCCTC
Cxcl11	AGGGCTCACAGTCAGACGTTCCCAGGATGTCACATGTTTTGACGCCTTAACTCCCCTTTCCCAAGTAAATGTACGGGAATTATCG	CGAAAGCCATGACCTCCGATCACTCATGTTCCAAGACAGCAGAGGGTCAGGTTCCTGGCACAGAGTTCTTATTGG
Cxcl9	GATGCCCAAGAGGAAAAGAACAGCGGACTTCATGGCAGAGCTGAGTTCTACTACGGTTACCGTCTTTATAAGTGAACAAAACCGG	CGAAAGCCATGACCTCCGATCACTCGCATTCCTTATCACTAGGGTTCCTCGAACTCCACACTGCTCCAGGAAGAT
Emr1	TGCAGACAGAATTCAGTCCACATTGTAATGGATCTTGGGTGCACTCATCACGAACCTAACTCCTCGCTACATTCCTATTGTTTTC	CGAAAGCCATGACCTCCGATCACTCCATTTGAAAGTCAGGGAGGCAGCCACAGATGTAGGAGCCTGGTACATTGG
Fcgr1	GGAGATCTGAACGGCTGTTCCGTTGATAAACCATTGTGTGGAACTGTCTCCAAAGACGCCTATCTTCCAGTTTGATCGGGAAACT	CGAAAGCCATGACCTCCGATCACTCTATTCGCCACTGTCCTGAAAACTGGCCTCTGGGATGCTATAACTAGGCGT

Flt3#	GGCAGACAGCATCTAGAGAAAAGTCCAGCGAAGCAACGGCCTGTAGGCCCGAAGCAATACTGTCGTCACTCTGTATGTCCGT	CGAAAGCCATGACCTCCGATCACTCCCACCTGTGCGAGGAGAGGTTTGATTTTATAGAAGTCACTTTGGAGTAAT
Gapdh*	ATCGAAGGTGGAAGAGTGGGAGTTGCTGTTGAAGTCGCAGGAGACAACCTCCTCAAGACCTAAGCGACAGCGTGACCTTGTTTCA	CGAAAGCCATGACCTCCGATCACTCCAGGAAATGAGCTTGACAAAGTTGTCATTGAGAGCAATGCCAGCCCCGGC
Gusb*	GTTCGTCATGAAGTCGGCGAAATTCCAGATGAGCTCTCCGACCACGTATTCATCCTCTTCTTTTCTTGGTGTTGAGAAGATGCTC	CGAAAGCCATGACCTCCGATCACTCTGGCGAGTGAAGATCCCCTTCTTGTTTCCGATTACTCTCAGCGGTGACTG
H2-Ab1	TCCCGTTGGTGAAGTAGCACTCGCCCATGAACTGGTACACGAAATGCCCAATTTGGTTTTACTCCCCTCGATTATGCGGAGT	CGAAAGCCATGACCTCCGATCACTCCCGGTTGTAGATGTATCTGGTCACATATCGTATGCGCTGCG
Hprt*	CTCCGGAAAGCAGTGAGGTAAGCCCAACGCTCTCCCACAATTCTGCGGGTTAGCAGGAAGGTTAGGGAAC	CGAAAGCCATGACCTCCGATCACTCCAAAAAGCGGTCTGAGGAGGAAGCCGGCGGAGGAGGTGCTACCG
Il10	TCTGAACTCAGGGATGAGAGCAATTGAAAGGACACCATAGCAAAGGGCCCCATAAAATTGGTTTTGCCTTTCAGCAATTCAACTT	CGAAAGCCATGACCTCCGATCACTCTGGTTTCTCTTCCCAAGACCCATGAGTTTCTTCACAACTCTCTTAGGAGC
Il10ra	TAGGCTCAGGCTGGAGATCGTGACGAGGAATGGGAGCAAACGCGACAACACTGGTCAAGACTTGCATGAGGACCCGCAAATTCCT	CGAAAGCCATGACCTCCGATCACTCAACCACACATAGGAAGGGCTTGGCAGTTCTGTCCCGTATGCAATGAATTC
Il1a	ACCCGGCTCTCCTTGAAGGTGAAGTTGGACATCTTTGACGTTTCAGAGGTCCTGAATCAATAGAACAATATCAGTTATGGCGGTG	CGAAAGCCATGACCTCCGATCACTCGCCGTCTCTTCTTCAGAATCTTCCCGTTGCTTGACGTTGCTGATACTGTC
Il1b	CAGCTAGGTTCTGTTCTAGAGAGTGCTGCCTAATGTCCCCTTGAATCAACCGGTTGTTAATATGACAGGCCGCTAAAGACGTTCT	CGAAAGCCATGACCTCCGATCACTCAATGAAAGACCTCAGTGCGGGCTATGACCAATTCATCCCCCACACGTTGA
Il23a	AAGCCAGACCTTGGCGGATCCTTTGCAAGCAGAACTGGCTGTTGTCCTTGCTTTCGTTGGGACGCTTGAAGCGCAAGTAGAAAAC	CGAAAGCCATGACCTCCGATCACTCTAGAGCAGGCTCCCCTTTGAAGATGTCAGAGTCAAGCAGGTGCTTATAAA
Il4#	CGAAAGAGTCTCTGCAGCTCCATGAGAACACTAGAGTTCTTCTTCAAGCACCGTCTCAGATGAGTGGGTTAATCAATCAAGTATG	CGAAAGCCATGACCTCCGATCACTCTGGACTCATTCATGGTGCAGCTTATCGATGAATCCAGGCATCGAAAAGCC
Il6	ACCAGCATCAGTCCCAAGAAGGCAACTGGATGGAAGTCTCTTGCCCAGCAGACCTGCAATATCAAAGTTATAAGCGCGT	CGAAAGCCATGACCTCCGATCACTCCTCTCCGGACTTGTGAAGTAGGGAAGGCCGTGGTTGTC
Irf4	GCAGGATCAGGACATGATAGCCTAGACTGGACCTAAGAACCAAGTCCCCTGGTCTAGGTATCTAATTCGTGGGTCGGGTACT	CGAAAGCCATGACCTCCGATCACTCAAGGGCAAGAAGAGGGAGCAAACAGGATGTAGGGTCTACTCAGAGAGAGT
Irf5	ACCTTAAAAGTTGGCTACCCTGGGGTAATTGGACTCCGTGGTACTAAAGGCATTAGCTCGGATGCTATCAGCTTGCGCCTATTAT	CGAAAGCCATGACCTCCGATCACTCCCCACAACACTCCTTGGCAGGTTTTGCTAGAAGAATTGGGGCA
Itgax	CACAAGCCAACAGCCAGGAAGGGTTGGTGGCAGCAGCAAGGCCTCAAGACCTAAGCGACAGCGTGACCTTGTTTCA	CGAAAGCCATGACCTCCGATCACTCCCTGTCAAGTATATATTCTCTCTGCATGTGTGGTGCACAGTAGGAC

Itgb7	GAGTCACAGTGGATGACAGGCTATCATAAGCATCCATGATGAGCTGCTTTCGGGTTATATCTATCATTTACTTGACACCCT	CGAAAGCCATGACCTCCGATCACTCATTCAAAGGAGATGCTGACTCCTGGTGGGAGTGGAGAGTGCTCAA
Ly6c1	GAAAGGCACTGACGGGTCTTTAGTTTCCTTCTTTGAGAGTCCTCAATCAACATCCTCTTCTTTTCTTGGTGTTGAGAAGATGCTC	CGAAAGCCATGACCTCCGATCACTCCTGATGTTAGGATCCCTGATTGGCACACCAGCAGGGCAGAAA
Mrc1	AGACAGGATTGTCGTTCAACCAAAGCCACTTCCCTTCAACATTTCGGAACCACCGTGTGGACGGCAACTCAGAGATAACGCATAT	CGAAAGCCATGACCTCCGATCACTCACAATCATTCCGTTCACCAGAGGGATCGCCTGTTTTCCAGTTGACAAAGG
Nfkb1	AGATCCCTCACAAGCTGAGCGTGGAGGTGGATGATGGCTAAGCTTACAGATCGTGTGCTCATGACTTCCACAGACGT	CGAAAGCCATGACCTCCGATCACTCTTCTCATGTTGATGATGTCATCAGAGATCAAACCAGATGTGACTTCCAGC
Nfkb2	CAGCTGCAGGAACACCGTTACAGGCCTCTCGATCTTCATCTTGTGATAGGCTTGGAGGAGTTGATAGTGGTAAAACAACATTAGC	CGAAAGCCATGACCTCCGATCACTCTAATATGTGAACTGTTTGGAGTCCGAGACATCGCCCCCACGCTTGCGTTT
Nod2	GTCCAAGCCATCAAAGGTTAACAGGACACGGTCAGGATGGTCAAGAAGGACACCAGTTAGCGTGGCGTATACCATGTTGTTAACA	CGAAAGCCATGACCTCCGATCACTCTGGCGCTCCCGGTCGGTGAACCGGAACTTGAACTC
Nos2#	TTAGTAGTCCACAATAGTACAATACTACTTGGTAGGGTGGAGGAGGGGGGCAAACTGGAGAGAGAAGTGAAGACGATTTAACCCA	CGAAAGCCATGACCTCCGATCACTCGGAGAAGAAGGGAGGAAAGGGAGAGAGGGGAGGGAGGAGAGGAGAGAGAT
Pparg#	CATCGTGTAGATGATCTCATGGACACCATACTTGAGCAGAGTCACTTGGTCCTACGTATATATCCAAGTGGTTATGTCCGACGGC	CGAAAGCCATGACCTCCGATCACTCCCCTCTGAGATGAGGACTCCATCTTTATTCATCAGGGAGGCCAG
Relb	CCCCTTTTCCTCTTCGGACTCAGCATTCTGTAAAATGGGCTCAAAGAGAACCGATCTTCATAACGGACAAACTGAACGGGCCATT	CGAAAGCCATGACCTCCGATCACTCACAATCTCTGAGAATCTGTCCTGAGAAGGGGTCCATCTGCAGGAG
Retnla	GTTCCTTGACCTTATTCTCCACGATAATCTCTATGGTCTCATCAGTATTCCTATCAGCTAATAGGGTCGGCTCAACAGTGTATCC	CGAAAGCCATGACCTCCGATCACTCAGAGAGAGTCTTCGTTACAGTGGAGGGATAGTTAGCTGGATTGGCAAGAA
Rpl19*	TTGGGATCCAACCAGACCTTCTTTTTCCCGCAGCGCTGTTGAGATTATTGAGCTTCATCATGACCAGAAG	CGAAAGCCATGACCTCCGATCACTCCTGACGGGAGTTGGCATTGGCGATTTCATTGGTCTCA
Sell	CAGTCCCAAGTAGCTCTCTTCCCTCAGAACAGTTGAAAGCTATCAATTCGTGACCCCGATCATCCAGTCCAGAA	CGAAAGCCATGACCTCCGATCACTCATGACCAGTTTCCAGATGCTCCACACTGTGTTTCTG
Siglec-1	TGTAGTCACCAGGAACTGGAGTGAGGAAGCCGGTCCAGATAAGGTTGTTATTGTGGAGGATGTTACTACA	CGAAAGCCATGACCTCCGATCACTCTGTATCATGCACCTGGCAAAAGTAAGCACCAGCGTGAGCCCG
Spi1	GTAAGTAACCAAGTCATCCGATGGAGGGGCGGTGAGGGAAAACCCTTCCACAAATGCACTCTATATGGAGGGAGAGTAGCTGGAT	CGAAAGCCATGACCTCCGATCACTCCCCACGAAGGAGTAGTAGTCATGCATTGGACGTTGGTATAGCTCTGAATC
Stat1	AAGTTCTTCGGTGACAATGAGAGGCCCCTCATTAGTTCTGTTCCCAGCGTCCTATGCATCATGTGCCTCACTAGGACATCATGCT	CGAAAGCCATGACCTCCGATCACTCTCAATCACCAAGCCTGGCTGGCACAACTGGGTTTCAAAGCTAAGAGAGTG
Stat3#	ATCTGGGTCTTGCCACTGATGTCCTTTTCCACCCAAGTGAAAGTGACCCACCCCTCCAAACGCATTCTTATTGGCAAATGGAA	CGAAAGCCATGACCTCCGATCACTCCAAATGACATGTTGTTCAGCTGCTGCTTGGTGTATGGCTCTACAGACTGG

Stat6	AGCCACATAGTTACTCCCCAAATGATACTGGGTTGCTGTCTTATACAATGCCTAAATTGGGAAAAAAGGTTTTAGCTATTGATGG	CGAAAGCCATGACCTCCGATCACTCGAAACACTGGAAGCATCAAGTTACCCAGGCTAGAGCTTTATGCATGTCAC
Tgfb1	CGAAAGCCCTGTATTCCGTCTCCTTGGTTCAGCCACTGCCTTCCTTCCTGTGTTCCAGCTACAAACTTAGAAAC	CGAAAGCCATGACCTCCGATCACTCCCACGTGGAGTTTGTTATCTTTGCTGTCACAAGAGCAGTGAGCGCTGAAT
Tlr1	TTCAAGCACACACTTGATGTTAGACAGTTCCAAACCGATCGTAGTGCTGACATGTTGGAGTTAACGGAGACCCGCCATCGTTTAC	CGAAAGCCATGACCTCCGATCACTCTTCTTTCCAAGCTTTGACAAAGCACGTAAGAAATAAGAGCAGCCCTGGTC
Tlr2#	ACCATCAGAAACTATGATTGCGGACACATCTCCTGCCAGTGACCGCCCACGATCTGTATTTTGCACCTTTCGCTATGCTGAG	CGAAAGCCATGACCTCCGATCACTCGAACGACATCTTTTCCACTTCTAGGTCGCAGAGACTGCCGTCCAACCTTC
Tlr4	ACTGGGTTTAGGCCCCAGAGTTTTGTTCTCCTCAGGTCCAAGTTGCTGTGTCCGTCTATACGCATACTGGTCCACATATA	CGAAAGCCATGACCTCCGATCACTCGTCCATAGCAGAGCCCCAGGTGAGCTGTAGCATTTATTAATTGCAAACAG
Tlr8	GTGCCTTATCTCGTCACAAGGATAGCTTCTGGAATAGTTCGCTTTATGCGCTCATTTTGAACATACGATTGCGATTACGGAAA	CGAAAGCCATGACCTCCGATCACTCGGAACTTCATGCAGTTGACGATGGTTGCATTCTGCAATCACAAGGGAGTT
Tnf	ATTCTGAGACAGAGGCAACCTGACCACTCTCCCTTTGCAGAACTCAGGAACCTGCCAATGCACTCGATCTTGTCATTTTTTTGCG	CGAAAGCCATGACCTCCGATCACTCCTGGAAAGGTCTGAAGGTAGGAAGGCCTGAGATCTTATCCAGCCTC
Traf6	ACAGCTTTGATCATGGATCTCTTTCTCTTCATATGCCATGGACACAGCGCTATGCAGACGAGCTGGCAGAGGAGAGAAATCA	CGAAAGCCATGACCTCCGATCACTCTCTCTGATGAGGATTGTACCACAGTATTCACAGATGATATTTGCCAGAGG
Trem1	GGTGGAAACAAGCAAATGCCAGTGTGTCCTAGAGAAAGCACAGGAGCCACCGATTGCTGCATTCCGCTCAACGCTTGAGGAAGTA	CGAAAGCCATGACCTCCGATCACTCCACTGCTGAAGTAGAAGGAATGGATCTACCATCCTTTTCCCACCCAAAGA
Trem2	AGGCCAGGAGGAGAAGAATGGAGGTGGGTGGGAAGGAGGTCTCTGAGGCTGTTAAAGCTGTAGCAACTCTTCCACGA	CGAAAGCCATGACCTCCGATCACTCCCACAGCCCAGAGGATGCTGGCTGCAAGAAACTTGCTCAGGAGAACGC
VEGFa	CTTCCGGGCTTGGCGATTTAGCAGCAGATATAAGAAAATGGCGAATCCAGCAACAGCCACTTTTTTTCCAAATTTTGCAAGAGCC	CGAAAGCCATGACCTCCGATCACTCTGTGTATGTGGGTGGGTGTGTCTACAGGAATCCCAGAAACAACCCTAAT
Zbtb46	AGACCCATAGCCTACATCTCCAGGCCACAAAGACTGTGAAGAAACGTCATCCGGGAATCGGCATTTCGCATTCTTAGGATCTAAA	CGAAAGCCATGACCTCCGATCACTCTCCCTCCATAATGTGATGGTGAAATCTGTTCTTCCTTGATGCGCAG

*Housekeeping genes

#genes excluded from the analysis because a general low detection level

Supplementary Table 3: List of antibody used

Antibody (Mouse)	Clone	Fluorochrome	Source	Catalog number	Dilution factor
CCR2	475301	PE	R&D	FAB5538P	1:200
CCR9	CW-1.2	AF647, PECy7	Biolegend	128708, 128711	1:100
CD103	M290	AF647, AF488	Biolegend	121410, 121408	1:200
CD115	AFS98	APC	Biolegend	135510	1:200
CD11b	M1/70	PE-594, APCCy7, BV605	Biolegend	101256, 101226, 101237	1:200
CD11c	N418	APCCy7, BV605	Biolegend	117324, 117334	1:200
CD172a	P84	PE	BD	560107	1:200
CD206	C068C2	AF647	Biolegend	141712	1:200
CD4	RM4-5	APCCy7	Biolegend	100526	1:200
CD45.1	A20	FITC, PE, AF647	Biolegend	110706, 110708, 110720	1:200
CD45.2	104	FITC, APC, APCCy7, AF700	Biolegend	109806, 109814, 109824	1:200
CD62L	MEL-14	APCCy7, BV711	Biolegend	104428, 104445	1:200
CD64	X54-5/7.1	PECy7, APC	Biolegend	139314, 139306	1:200
CX3CR1	SA011F11	FITC, PE	Biolegend	149020, 149006	1:200
Int-a4b7	DATK32	PE, APC	Biolegend	120606, 120608	1:200
Ly6C	HK1.4	PerCPCy5.5, BV711	Ebioscience, Biolegend	45-5932, 128037	1:400
Ly6G	1A8	PE, BV711	Biolegend	127607, 127643	1:200
MHCII	M5/114.15 .2	BV510, AF700	Biolegend	107635, 107622	1:200
Siglec F	E50-2440	PE	Biolegend	552126	1:200
DAPI			Carl Roth	6843.1	1:1000
7AAD			Biolegend	420404	1:20
