# Supplementary material for: Inflammation triggers immediate rather than progressive changes in monocyte differentiation in the small intestine
Source: Nat Commun. 2019 Jul 19;10:3229. doi: 10.1038/s41467-019-11148-2 (PMC6642215; doi:10.1038/s41467-019-11148-2)
Supplement: Supplementary file 3 — Reporting Summary [file 41467_2019_11148_MOESM3_ESM.pdf]

nature research

Corresponding author(s): Oliver Pabst

Last updated by author(s): Jun 15, 2019

Reporting Summary

Nature Research wishes to improve the reproducibility of the work that we publish. This form provides structure for consistency and transparency in reporting. For further information on Nature Research policies, see Authors & Referees and the Editorial Policy Checklist.

Statistics

For all statistical analyses, confirm that the following items are present in the figure legend, table legend, main text, or Methods section.

n/a	Confirmed	
☐	☒	The exact sample size (*n*) for each experimental group/condition, given as a discrete number and unit of measurement
☐	☒	A statement on whether measurements were taken from distinct samples or whether the same sample was measured repeatedly
☐	☒	The statistical test(s) used AND whether they are one- or two-sided<br>*Only common tests should be described solely by name; describe more complex techniques in the Methods section.*
☐	☒	A description of all covariates tested
☐	☒	A description of any assumptions or corrections, such as tests of normality and adjustment for multiple comparisons
☐	☒	A full description of the statistical parameters including central tendency (e.g. means) or other basic estimates (e.g. regression coefficient) AND variation (e.g. standard deviation) or associated estimates of uncertainty (e.g. confidence intervals)
☐	☒	For null hypothesis testing, the test statistic (e.g. *F*, *t*, *r*) with confidence intervals, effect sizes, degrees of freedom and *P* value noted<br>*Give P values as exact values whenever suitable.*
☒	☐	For Bayesian analysis, information on the choice of priors and Markov chain Monte Carlo settings
☐	☒	For hierarchical and complex designs, identification of the appropriate level for tests and full reporting of outcomes
☒	☐	Estimates of effect sizes (e.g. Cohen's *d*, Pearson's *r*), indicating how they were calculated

Our web collection on statistics for biologists contains articles on many of the points above.

Software and code

Policy information about availability of computer code

Data collection	not applicable
Data analysis	not applicable

For manuscripts utilizing custom algorithms or software that are central to the research but not yet described in published literature, software must be made available to editors/reviewers. We strongly encourage code deposition in a community repository (e.g. GitHub). See the Nature Research guidelines for submitting code & software for further information.

Data

Policy information about availability of data

All manuscripts must include a data availability statement. This statement should provide the following information, where applicable:

- Accession codes, unique identifiers, or web links for publicly available datasets
- A list of figures that have associated raw data
- A description of any restrictions on data availability

accession codes are given in the manuscipt, a raw data file as been submitted. All other data generated during and/or analysed during the current study are available from the corresponding author on reasonable request.

Field-specific reporting

Please select the one below that is the best fit for your research. If you are not sure, read the appropriate sections before making your selection.

☒ Life sciences ☐ Behavioural & social sciences ☐ Ecological, evolutionary & environmental sciences

Life sciences study design

All studies must disclose on these points even when the disclosure is negative.

Sample size	The relevant samples size relates to the number of mice analyzed. In all experiments individual animals were analyzed. Experiments were designed to inlcude multiple mice per group and to include independent multiple independent experiments. The number of animals and experiments is stated in the figure legends. Samples size was choosen based on previous experiments.
Data exclusions	No data were excluded from the analysis.
Replication	All experiments were independently repeated. The number of repeat experiments and the number of animals analyzed per experiment are stated in the figure legends
Randomization	Mice were randomly assigned to experimental groups
Blinding	Data were not blinded. No subjective scoring was used in the study.

Reporting for specific materials, systems and methods

We require information from authors about some types of materials, experimental systems and methods used in many studies. Here, indicate whether each material, system or method listed is relevant to your study. If you are not sure if a list item applies to your research, read the appropriate section before selecting a response.

Materials & experimental systems

n/a	Involved in the study	
☐	☒	Antibodies
☒	☐	Eukaryotic cell lines
☒	☐	Palaeontology
☐	☒	Animals and other organisms
☒	☐	Human research participants
☒	☐	Clinical data

Methods

n/a	Involved in the study	
☒	☐	ChIP-seq
☐	☒	Flow cytometry
☒	☐	MRI-based neuroimaging

Antibodies

Antibodies used	Fluorochrome-conjugated monoclonal antibodies specific to CCR9 (CW-1.2), CD103 (M290), CD115 (AFS98), CD11b (M1/70), CD11c (N418), CD206 (C068C2), CD4 (RM4-5), CD45.2 (104), CD45.1 (A20), CD62L (MEL-14), CD64 (X54-5/7.1), Ly6C (HK1.4), MHCII (M5/114.15.2), CX3CR1 (SA011F11), Ly6G (1A8), Siglec F (E50-2440), α4β7 (DATK32) from Biolegend; CCR2 (475301),and TREM1 (174031) from R&D; Ly6C (HK1.4) from ebioscience; CD172a (P84) from BD as well as live/dead staining DAPI (from Carl Roth) or 7AAD (Biolegend) were used in these experiments.
Validation	All antibodies used is this study were commercially available and validation statement is on the manufacture's website.

Animals and other organisms

Policy information about studies involving animals; ARRIVE guidelines recommended for reporting animal research

Laboratory animals	C57BL/6, CX3CR1gfp/+, CCR2-/-, Itgb7-/-, CCR9-/- mice were bred and maintained on C57BL/6 background under specific-pathogen free conditions. Both male and femal adult mice were used in the present study
Wild animals	not applicable
Field-collected samples	not applicable
Ethics oversight	The study protocol and all procedures in the laboratory mice were performed in accordance with local guidelines and regulations of Animal Protection Act (Landesamt fur Natur, Umwelt und Verbraucherschutz Nordrhein-Westfalen, LANUV) or the Niedersächsisches Landesamt für Verbraucherschutz und Lebensmittelsicherheit (LAVES)

Note that full information on the approval of the study protocol must also be provided in the manuscript.

Flow Cytometry

Plots

Confirm that:

☒ The axis labels state the marker and fluorochrome used (e.g. CD4-FITC).

☒ The axis scales are clearly visible. Include numbers along axes only for bottom left plot of group (a 'group' is an analysis of identical markers).

☒ All plots are contour plots with outliers or pseudocolor plots.

☒ A numerical value for number of cells or percentage (with statistics) is provided.

Methodology

Sample preparation	Cells isolated from small and large intestinal lamina propria, liver, spleen, bone marrow and single cell suspensions were prepared for flow cytometry acquisition and/or sort.
Instrument	BD FACS LSR Fortessa and Ariall Sorter
Software	FCAS Diva and FlowJo VX softwares were used for cell acquisition and analysis respectively.
Cell population abundance	Cells sorted were re-acquired for purity and was more than 95% in the experiments.
Gating strategy	First single leukocytes were defined based on the FSC/SSC gates of the starting cell population and then specific gating on defined populations are made as indicated in the figures. Gating strategies are shown in Supplementary Figure 8.

☒ Tick this box to confirm that a figure exemplifying the gating strategy is provided in the Supplementary Information.
